# Microfluidic Purification and Concentration of Malignant Pleural Effusions for Improved Molecular and Cytomorphological Diagnostics

**DOI:** 10.1371/journal.pone.0078194

**Published:** 2013-10-28

**Authors:** James Che, Albert J. Mach, Derek E. Go, Ish Talati, Yong Ying, Jianyu Rao, Rajan P. Kulkarni, Dino Di Carlo

**Affiliations:** 1 Department of Bioengineering, University of California Los Angeles, Los Angeles, California, United States of America; 2 Department of Pathology and Laboratory Medicine, UCLA Medical Center, Los Angeles, California, United States of America; 3 Division of Dermatology, UCLA Medical Center, Los Angeles, California, United States of America; University of Illinois at Chicago, United States of America

## Abstract

Evaluation of pleural fluids for metastatic cells is a key component of diagnostic cytopathology. However, a large background of smaller leukocytes and/or erythrocytes can make accurate diagnosis difficult and reduce specificity in identification of mutations of interest for targeted anti-cancer therapies. Here, we describe an automated microfluidic system (Centrifuge Chip) which employs microscale vortices for the size-based isolation and concentration of cancer cells and mesothelial cells from a background of blood cells. We are able to process non-diluted pleural fluids at 6 mL/min and enrich target cells significantly over the background; we achieved improved purity in all patient samples analyzed. The resulting isolated and viable cells are readily available for immunostaining, cytological analysis, and detection of gene mutations. To demonstrate the utility towards aiding companion diagnostics, we also show improved detection accuracy of KRAS gene mutations in lung cancer cells processed using the Centrifuge Chip, leading to an increase in the area under the curve (AUC) of the receiver operating characteristic from 0.90 to 0.99. The Centrifuge Chip allows for rapid concentration and processing of large volumes of bodily fluid samples for improved cytological diagnosis and purification of cells of interest for genetic testing, which will be helpful for enhancing diagnostic accuracy.

## Introduction

The pleural space surrounds the lungs and is lined by the pleural sac. Under certain conditions, including malignancies, this space can fill with excess fluid, resulting in a pleural effusion. Thoracentesis is a procedure to remove this pleural fluid, both for diagnostic and therapeutic purposes. Over 1.5 million thoracentesis procedures are conducted annually in the United States [1]. Many cell types may be present within the pleural effusion, and identifying and isolating these cells is important to identify the ongoing disease process. Cytologists analyze pleural samples to determine the cause (presence or absence of cancer) by examining stained cell smears on a glass slide. Sample preparation and analysis requires technician-intensive sample handling involving multiple centrifugation steps followed by staining and time-consuming manual microscopic scanning of cytology slides by the cytopathologist, who must search for key cancer cell morphological characteristics, such as high nuclear-to-cytoplasmic ratios, hypochromatic cytoplasms, and dense, dark nuclei.

Disseminated cancer cells originating from the lung, breast, or other organs can be identified in malignant pleural effusions. Traditional cytomorphological analysis of cell smears and blocks has high specificity, but low sensitivity. The low sensitivity can be either due to the subjective nature of analysis, loss of tumor cells during processing, or the fact that there may be few tumor cells present in a large specimen volume with obscuring blood. In up to 40% of cases, traditional cytological examinations may fail to identify malignant cells [2]. Therefore, approaches to obtaining these malignant cells from larger volumes of fluid with high purity and efficiency could improve cytology-based diagnoses [3]. Additional applications for purified cells from pleural and other body fluids include the ability to probe cellular properties such as cell deformability [4], [5], evaluation of effusion microenvironments [6], and identifying cellular metastases [7].

Tumor cells present in effusions may also be the only easily accessible source of malignant cells following cancer relapse, particularly in cases where the original mass was removed. Isolating cancer cells within malignant effusions may therefore be critical in order to perform molecular analysis or other tests to determine the up-to-date mutational status of the cancer that is crucial for the determination of targeted therapy [8]. Previous studies have indicated that only a subset of pleural effusions are suitable for mutational analysis because of the presence of large quantities of contaminating cells; malignant pleural fluids are almost always bloody, with large populations of leukocytes and/or erythrocytes [9]. Specifically, the leukocytes contain wild type DNA that may interfere with the detection of genetic mutations of interest [10].

Increasing sample purity enables improved molecular diagnostics to detect the presence of specific genetic mutations which may be amenable to targeted therapies. This can be achieved by removing a large population of leukocytes that contain interfering wild-type DNA. For genetic testing such as quantitative PCR (qPCR), the presence of a small quantity of mutated genes can be overshadowed by a large background of wild type nucleic acids. Using qPCR, the cycle threshold (Ct) gives a relative measurement of the amount of genetic material of interest that is present; a lower Ct indicates a greater amount of the gene of interest. Although qPCR can be exquisitely sensitive for mutation detection given appropriate selection of amplification primers, there is often some non-specific amplification from background DNA. The presence of large quantities of background DNA can thus interfere with accurate measurement of the Ct due to this non-specific amplification; this effect may still be notable even after normalization with housekeeping genes.

There are several approaches that are currently utilized to isolate cells of interest from pleural effusions for molecular analysis. The gold standard is laser capture microdissection (LCM), a technique used to isolate pure populations from cytology fluids, live cell culture, or heterogeneous tissue sections [11], [12], [13], [14], [15]. However, this technique requires drying out of cells during capture, which can lead to cell damage and is not capable of extracting large quantities of cells for analysis. It is also very time and labor intensive. Flow cytometry and fluorescence activated cell sorting (FACS) are also common methods for cell separation and sorting. While FACS can process samples of up to 30 mL in 1 hr, the sorted cells may not be suitable for further analysis as a result of the initial fixing and cell type-specific staining required for the sorting process. Microfluidic technology is an emerging tool that may deliver automated, well-controlled platforms to purify target cells with the highest possible sensitivity and specificity. Several strategies have been used to isolate and enrich tumor cells in body fluid [16], such as the use of self-assembled magnetic beads coated with anti-CD19 antibodies to capture B-cell malignant tumors [17]. However, current technologies are limited by throughput and purity, and none have been placed in widespread use in clinical labs for a variety of reasons. Many devices also focus on rare cell isolation from blood rather than tumor cell enrichment from pleural effusions, which have unique fluid properties and cellular profiles. Ideally, rapid sampling of pleural fluids (often liters of fluid) requires mL/min processing rates and separation using a label-free marker such as cell size [18]. Moreover, sample preparation of pleural effusions should be performed in an automated, repeatable fashion to enable clinicians and cytopathologists to perform molecular assays on the purified cells with the highest possible sensitivity and specificity in a short time period (tens of minutes).

**Figure 1:**
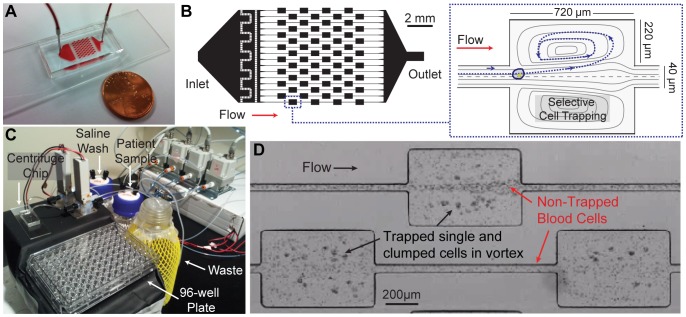
Principles of the Centrifuge Chip. (A) A photograph of the Centrifuge Chip device. Only a single inlet and outlet is required. (B) A schematic of the massively parallel microfluidic device that selectively traps large cells in individual microscale vortices. (C) A photograph of the device connected to an automated fluidic instrument to deliver patient pleural samples and saline wash through the Centrifuge Chip into the waste bottle or collection plate. Trapped epithelial and mesothelial cells are made readily available i) into a collection tube for further cytology slide comparisons with the original and/or ii) into a well-plate for immunolabeling, imaging and analysis. (D) High-speed microscopic image showing trapping of larger single and clumped cells while smaller red and white blood cells pass through.

We have previously demonstrated a potentially low cost, miniaturized microfluidic system that recapitulates the high-throughput operations of enrichment and concentration of a standard laboratory centrifuge (the “Centrifuge Chip”) [19]. Here, we use the Centrifuge Chip for the isolation of cancer cells and mesothelial cells at high purity from pleural effusions as a preparation step for downstream analysis by traditional cytology and mutational analysis (**Figure 1A**). By processing a large volume of fluid and selectively enriching larger cells over a background of red and white blood cells we replace the traditional centrifugation step in the clinical lab while also potentially enabling more sensitive analysis of purer preparations originating from large volume samples. Briefly, the approach employs unique inertial fluid physics to selectively collect larger cells (such as tumor cells) in laminar fluid microvortices at high rates without clog-prone filters [19], [20] (**Figure 1B** **, Video S1**). Smaller leukocytes and erythrocytes are not stably trapped in vortices and are significantly reduced in the collected concentrated sample (**Figure 1D**). We have also implemented fluid plumbing automation to process samples and release isolated cells back into a small volume, under the control of a custom-written software program **(** **Figure 1C** **, Figure S1**). Each Centrifuge Chip processes effusions at a flow rate of 6 mL/min and concentrates larger cells (mesothelial and epithelial). Purified cells are released and made readily available in a collection vial or micro-titer plate for cytology and identifying gene mutations.

## Materials and Methods

### Ethics Statement

The study is exempt from institutional review board approval because remnants of patient specimens were processed and analyzed anonymously, with no access to protected health information, personal identifying information, or sensitive information. The study is exempt under 45 CFR 46.101 Category 4, which includes “Research involving the collection or study of existing data, documents, records, pathological specimens, or diagnostic specimens, if these sources are publicly available or if the information is recorded by the investigator in such a manner that subjects cannot be identified, directly or through identifiers linked to the subjects.”

(http://www.hhs.gov/ohrp/humansubjects/guidance/45cfr46.html#46.101)

### Microfluidic Device Fabrication and Setup

Devices were fabricated using standard photolithography and polydimethylsiloxane replica molding techniques. The devices were designed in AutoCAD (Autodesk) and printed on a transparency photomask at 20,000 dots per inch (CAD/Art Service, Inc.). The mold was photolithographically defined using this mask in the UCLA Nanoelectronics Research Facility. Negative photoresist, KMPR 1050 (MicroChem), was spun at 2400 rpm for 30 s on a 10-cm silicon wafer. The wafer was soft-baked at 100°C for 15 min, exposed under near UV for 30 s, post-baked at 100°C for 4 min, and developed in SU-8 Developer (MicroChem). The height of the resulting feature was measured to be 55 µm using a profilometer (Veeco Metrology). Polydimethylsiloxane (PDMS) (Sylgard 184 Dow Corning Corp.) was poured onto the photoresist master at a 10∶1 ratio of base to crosslinker, degassed in a vacuum chamber, and cured at 65°C overnight. The devices were then cut from the mold, ports were punched with a punch kit (Technical Innovations), and the devices were bonded to glass slides using oxygen plasma for 30 s (Harrick Plasma). After plasma treatment and placement onto the glass substrate, the devices were maintained at 65°C in an oven for 15 min to increase bonding.

### Cell Trapping Mechanism

The mechanism of operation is based on size-dependent inertial lift, which leads to selective entry and stable orbits for larger cells within vortices created in an expansion reservoir [19], [20]. Smaller cells do not experience sufficient lift force and therefore either do not enter the vortex, or do not have enough restoring lift force to remain stable within the vortices in the presence of de-stabilizing disturbances from other orbiting particles. In our previous work we identified reservoir geometries and flow conditions to selectively collect cells and particles above ∼15 µm, with capture efficiencies of ∼20% for MCF7 cells spiked in diluted blood [19]. The rectangular reservoirs are 480 µm wide and 720 µm long, and the straight channels are 40 µm wide. In this work, we made several device modifications including 1) the integration with a custom-made pressure system that operates using a simple ‘plug-and-play’ option in which an operator does not need to be present at all times, 2) the shortening of the device channel length to reduce fluidic resistance, and 3) the increase of the number of parallel channels to 16 with 4 chambers in each channel for a total of 128 cell trapping reservoirs to process samples at a flow rate of 6 mL/min. At this flow rate one patient sample (∼50 ml of volume) takes <10 minutes to process. The capture efficiency of the device was ∼47%, which was defined as the number of 20 µm diameter beads caught and released from the vortices divided by the total number of beads injected.

### Sample Processing using a Computer-Controlled Pressure System

The device is connected to a custom-made pressure system that delivers effusion samples or saline wash from pressurized glass bottles through the Centrifuge Chip (**Figure S1**). The Labview-controlled system contains a pair of air regulators, air valves and liquid valves (SMC Corporation) that brings compressed air into the bottles and drives fluid through the microchip device. Non-diluted pleural effusion samples are poured directly into the glass bottle and introduced through the device at 6 mL/min. Once the vortex traps are filled with cells, PBS is introduced into the device to wash out untrapped blood cells in the main flow and the vortex traps. Cells trapped in the fluid vortex are released by reducing the input air pressure and subsequently lowering the flow rate and dissipating the vortex. We implement a ‘trap-and-release’ program that can continuously introduce sample through the Centrifuge Chip, wash, and release the captured cells in a small 250 µL volume into a microtiter plate or collection vial. A video of sample processing was recorded using Phantom Camera Control and Software (Vision Research Inc.) with a high-speed camera (Phantom v7.3).

### Sample Collection and Preparation

Remnants of 115 pleural effusion samples obtained from Ronald Reagan UCLA Medical Center, Santa Monica UCLA Medical Center, and Northridge Hospital Medical Center were used in our study. From all specimens, up to 50 mL of sample were processed with the Centrifuge Chip. Effusions were passed through a 40 µm cell strainer before introducing through the Centrifuge Chip system. Half of the processed samples were returned to the cytology laboratory to create cell smears. This was performed in parallel with cell smears produced with traditional cytological methods on original, unprocessed samples. The other half of processed samples were fluorescently labeled to quantify sample purity (**Figure S2**). A fraction of samples were profiled for cell size distributions before and after processing.

### Cell Smear Preparation and Imaging

Smears were prepared according to normal methods to prepare samples for clinical evaluation. Briefly, samples were aliquoted into 50 mL conical tubes and centrifuged down with a standard benchtop centrifuge. After centrifugation, the supernatant is aspirated and the cells are resuspended in a buffer solution and placed with a glass slide into a cytocentrifuge (Thermo Scientific) to create a cell smear. The cell slides are air dried or fixed and stained with Papanicolou (Pap) or May-Grunwald-Giemsa (MGG) stains.

### Fluorescent Staining for Purity Measurements

For each specimen, 300 µL of the original effusion was transferred into one well of a 96-well microtiter plate. To compare the processed sample versus the original sample, up to 10 mL effusion volume was processed with the Centrifuge Chip and isolated cells were released in a volume of ∼250 microliters in the microtiter plate. To determine the composition of the cell population, leukocyte, epithelial and nuclear stains were used. After centrifuging the cells to the bottom of the well with a plate centrifuge (Beckman Coulter), the supernatant was aspirated. Cells were treated with 4% v/v formaldehyde for 15 min, permeabilized with 0.4% v/v Triton X-100 (Sigma-Aldrich) for 7 min, and incubated with cytokeratin (CK)-PE (epithelial and mesothelial cells), CD45-FITC (white blood cells), and DAPI (nucleus) (Invitrogen) in 2% w/v BSA. Between each step, cells were sedimented with the centrifuge and washed with PBS. After staining, the cells were imaged using a CCD camera (Photometrics CoolSNAP HQ2) mounted on a Nikon Eclipse Ti microscope. The whole well was automatically imaged in a few minutes (100X) using an ASI motorized stage operated with Nikon NIS-Elements AR 3.2 software. Captured images were automatically obtained for four configurations: brightfield, FITC, TRITC and DAPI filter sets. Collected images were automatically stitched together using the NIS-Elements Software. Images were analyzed by enumerating the number of CK+ and CD45+ cells present in each well. Purity is defined as the number of CK+ cells divided by the total number of nucleated cells. CK+ cells include carcinoma cells and mesothelial cells. We did not attempt to separate tumor cells from mesothelial cells as these cells share a similar size, but these separations can be carried out using IHC markers such as Calretinin [21], if necessary, to further enrich a specimen.

### Quantification of Cell Size

Dilute volumes of unprocessed and processed pleural samples were lysed with red blood cell lysis buffer (Roche) and incubated with Calcein AM (Invitrogen) for 15 minutes. Cells were imaged using a Nikon Eclipse Ti fluorescent microscope, and cell sizes were automatically measured using Nikon NIS-Elements AR 3.2 software.

### Detection of KRAS Gene Mutations in Spiked Pleural Effusion Samples

We evaluated the performance of the Centrifuge Chip to improve the accuracy of mutational analysis by extracting molecular information from spiked pleural effusions before and after enrichment to determine the potential improvement to qPCR measurement provided by high purity capture. Initial cell concentration was quantified from a small pleural sample aliquot using a hemacytometer after performing a red blood cell lysis step. A549 lung cancer cells (ATCC) were spiked at 0.1% purity in 50 mL of pleural effusions diagnosed as negative for malignancy.

Spiked samples were evaluated for a known activating mutation in KRAS found in A549 cells, which can provide resistance to targeted therapies [11]. Specifically, we looked at the 34 G>A substitution in KRAS (KRAS*) as identified by the Sanger Cosmic database [22]. We utilized quantitative RT-PCR to identify mutant KRAS in the A549 cells versus wild type (in HeLa and other cells) using a modification of the system described by Morlan et al. (2009) [23]. We used a primer complementary to the mutant and a blocking primer with a nonhydrolyzable phosphate group complementary to the wild type sequence which was present at four times the concentration. The rationale for this strategy was that the non-hydrolyzable primer would block non-specific amplification from the wild type sequence while still allowing amplification from the mutant of interest. GAPDH mRNA was also amplified as an indicator for the relative number of cells in a given sample and used to normalize each measurement to determine the ΔCt value.

Briefly, reverse transcription was performed using a SuperScript III RT kit (Invitrogen) according to the manufacturer's instructions to create cDNA libraries. TaqMan PCR was performed using 2 uL of the RT product in a 20 uL total volume with 1× TaqMan Universal PCR Master Mix (no UNG) (Roche) with the primers at 900 nM, TaqMan probe at 200 nM, and blocker at 3600 nM. Stock TaqMan probes for GAPDH and KRAS were obtained from Applied Biosystems and used without modification. The thermocycling conditions were as follows: 10 minutes at 95°C, 40 cycles of 20 seconds at 95°C and 1 minute at 60°C.

## Results and Discussion

### Measurement of Cell Size Distributions in Pleural Effusions

**Figure 2:**
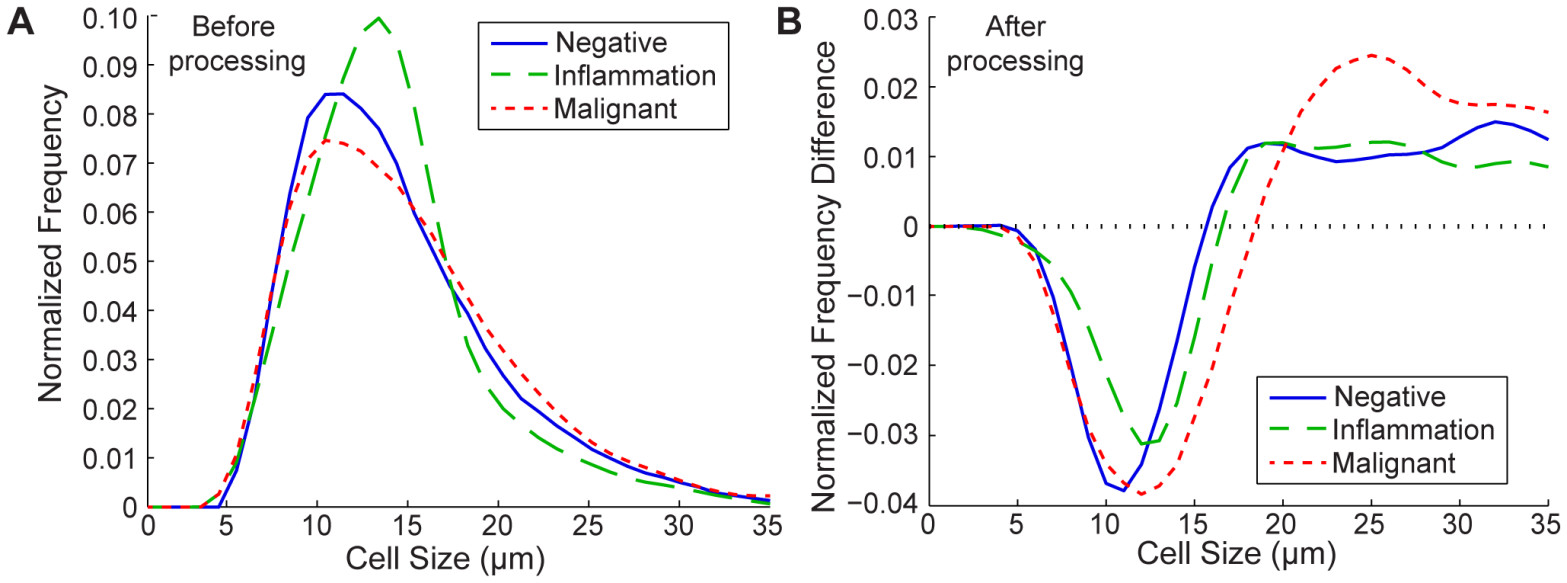
Distribution of Cell Size in Pleural Fluids and Enrichment with the Centrifuge Chip. (A) Normalized frequency distributions of nucleated cells in relation to cell diameter are plotted for pleural effusions analyzed within 24 hours of collection and diagnosed as negative (blue, solid), inflammation (chronic and acute, green, dashed), or malignant (red, dotted). Relative to negative pleural effusions, malignant samples contain a larger population fraction of cells greater than 15 µm, and cases of inflammation contain a larger population of cells between 10–15 µm. N = 13, 9, and 10 for negative, inflammation, and malignant cases. (B) The frequency distributions of the same samples after processing through the Centrifuge Chip were subtracted by the distributions before processing to observe enrichment. The device enriches for cell populations greater than 15 µm and depletes smaller cells.

Our system operates by selectively isolating cells above a particular size threshold [19], [20]. Therefore, we first measured detailed information on the number and diameter of cells present in 25 pleural fluid samples (**Figure 2**). Possible cytological diagnoses for pleural effusions included: positive for malignancy, suspicious or equivocal (atypical) for malignancy, and negative for malignancy (**Table 1**). Patient samples diagnosed as negative for malignancy were often concurrently diagnosed with acute inflammation, chronic inflammation, or lymphocytosis. Cytologically, samples with acute inflammation were associated with an increased neutrophil population; those with chronic inflammation were associated with a larger fraction of lymphocytes and histiocytes, while those with lymphocytosis were associated with increased lymphocytes. In cases positive for malignancy, the tissue of origin was often known from patient history.

**Table 1:** Summary of 115 Pleural Samples Analyzed in the Study.

Cytodiagnosis	Classification	Total Cases
Positive		25
	Lung	10
	Breast	6
	Ovarian	3
	Gastric	2
	Pancreatic	2
	Esophageal	1
	Mesothelioma	1
Suspicious		13
Negative		77
	Chronic Inflammation	25
	Lymphocytosis	15
	Reactive Changes	10
	Acute Inflammation	9
TOTAL		115

25/115 (21.7%) of samples were diagnosed positive for malignancy, 13/115 (11.3%) were diagnosed suspicious for malignancy, and 77/115 (67.0%) were diagnosed negative for malignancy.

We observed a population of cells greater than 15 µm in malignant samples (**Figure 2A**), consistent with the observation that malignant and mesothelial cells are usually larger compared to other cells present within these fluids [24]. Cases of inflammation had a large population of 10–15 µm cells, potentially representing the characteristic population of large activated immune cells. Of the samples diagnosed as Positive for Malignancy, on average 36.57% of nucleated cells were larger than 15 µm. A lower percentage of larger cells was present in samples diagnosed as Negative and Negative with Inflammation (32.47% and 26.92% of nucleated cells larger than 15 µm, respectively). Cases with inflammation are known to have a larger number of white blood cells as a fraction of the population, thus leading to a lower relative percentage of larger cells than negative samples alone. Note that these relatively large percentages of larger cells in non-malignant samples are likely the result of the presence of mesothelial cells and large activated leukocytes. Still, malignant samples contain the largest fraction of large cells such that cell size is a potential biomarker for harvesting malignant cells from pleural fluid samples. The Centrifuge Chip enriches for the cell populations greater than 15 µm (**Figure 2B**).

### The Centrifuge Chip Increases Purity of Clinical Samples

**Figure 3:**
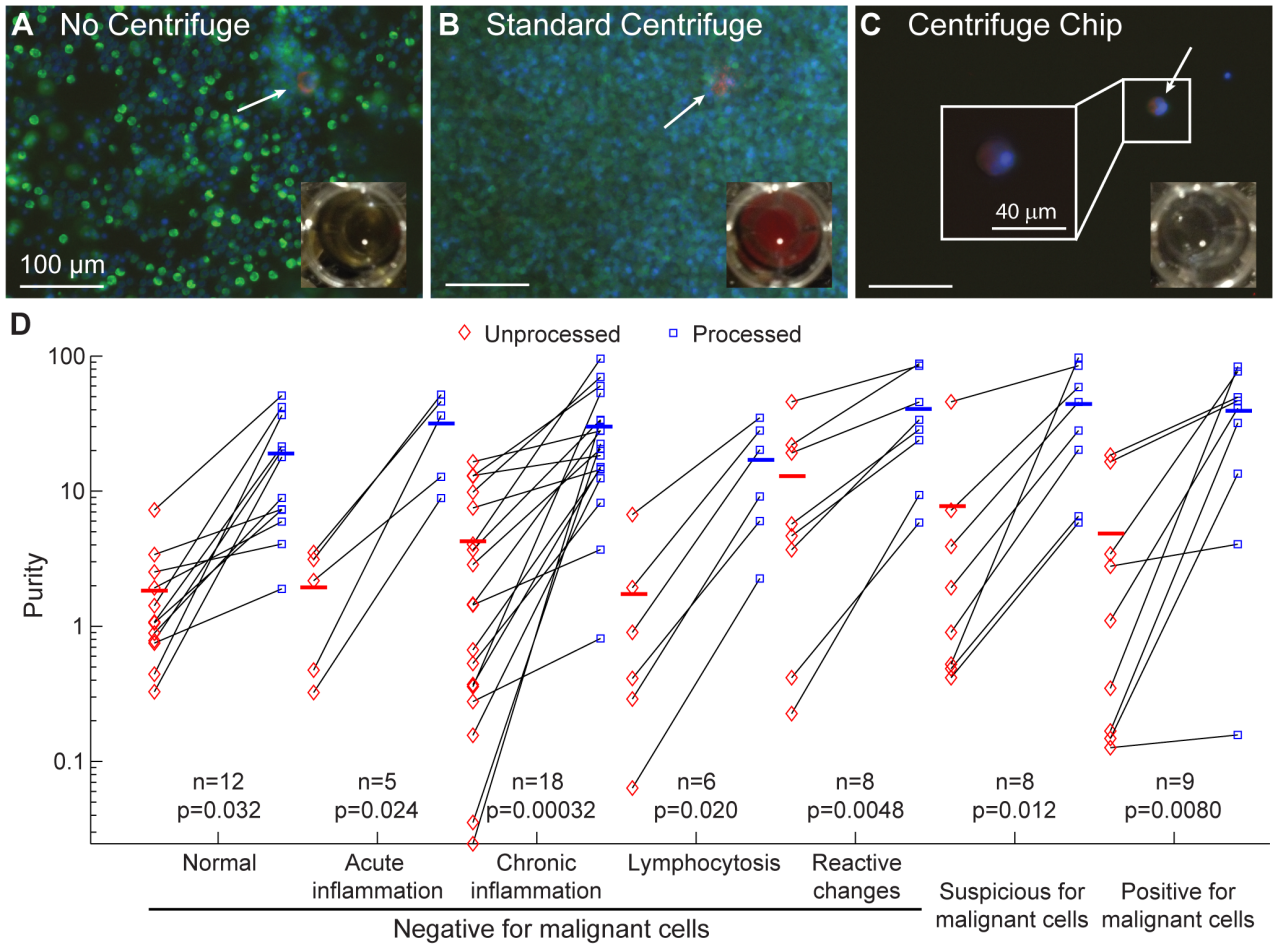
Paired Samples Following Centrifuge Chip Processing Show Purity Increases. (A–C) Qualitative comparison of unprocessed sample, centrifuged sample, and sample processed with the Centrifuge Chip. Immunofluorescent images of Cytokeratin (CK, red – epithelial cells), CD45 (green – leukocytes), and DAPI (blue – nucleus). Insets show well-plate color: yellow (fresh specimen), red (indicating bloody) and colorless (after processing with device). Arrows indicate CK+/DAPI+ epithelial cells. (D) Purity increases upon processing with the Centrifuge Chip. Purity is defined as the presence of CK+/DAPI+ (epithelial and mesothelial cells) over the total number of cells. All samples (N = 66) were seen to increase in purity after processing with the Centrifuge Chip. Red and blue bars represent the average purity.

Qualitatively, the Centrifuge Chip delivered a higher purity sample compared to unprocessed or centrifuged specimens (**Figure 3A–C**). The device increased purity in all 66 cases examined (100%) (**Figure 3D** **, Table S1**). Paired t-tests between unprocessed and processed samples demonstrated a significant increase in purity, with p values less than 0.05 for all diagnoses.

In agreement with our cell size measurements, we observed many cells captured for malignancy-positive cases and fewer for malignancy-negative cases with lymphocytosis, reactive changes, or acute inflammation. Additionally, the purity increased from unprocessed to Centrifuge Chip processed specimens (**Table 2**). Purity fold is defined as the purity of a chip-concentrated sample over the initial unprocessed sample. Greater than 65-fold increase was observed for samples diagnosed as positive for malignancy. Interestingly, samples with chronic inflammation had a 132-fold increase as a result of the larger leukocyte populations in the initial samples with <1% purity. Higher purities can be achieved by increasing the critical size cutoff of the Centrifuge Chip to further reduce leukocyte capture, although at the cost of potentially not trapping smaller malignant cells of interest.

**Table 2:** Purity Increases Following Processing with the Centrifuge Chip.

Cytodiagnosis	Purity fold increase
Positive	65.38
Suspicious	34.05
Negative	20.46
Lymphocytosis	20.66
Acute Inflammation	27.93
Chronic Inflammation	132.03
Reactive Changes	10.39

Purity fold is defined as the ratio of purities between a specimen processed with the Centrifuge Chip and the unprocessed specimen.

### Reduced Nonspecific Background from Cytology Slides and Reduced Sample Area

**Figure 4:**
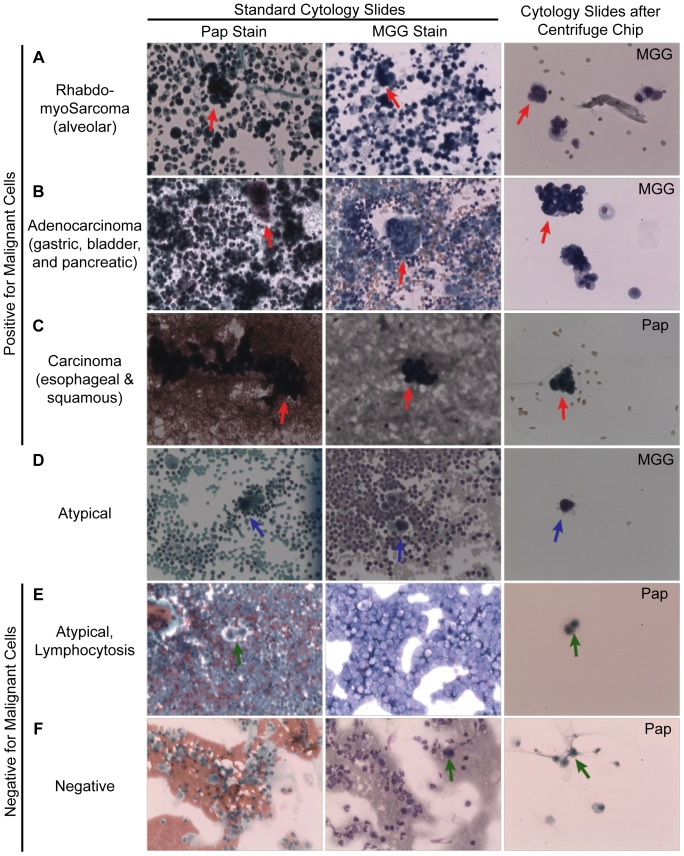
Reduced Background Cytology Slides When Prepared Using the Centrifuge Chip. Unprocessed patient samples are prepared with traditional cytological techniques using Papanicolaou (Pap) and May-Grunwald–Giemsa (MGG) stains. Cells collected from samples processed with Centrifuge Chip were also made into cytology slides. In all samples, malignant and mesothelial cells are found amongst a cellular background of immune cells in standard slides while little background is observed in the Centrifuge Chip slide. In samples diagnosed as ‘positive for malignancy’, single and clumped malignant cells (red arrows) are retrieved following the Centrifuge Chip. In patients diagnosed as ‘negative for malignancy’, mesothelial cells (green arrows) were found amongst a background of blood cells in standard slides, compared to no blood cell background upon Centrifuge Chip Processing. Samples diagnosed as atypical contained cells which resembled either malignant or mesothelial cells (blue arrows). Images obtained at 200× magnification.

We addressed issues of reducing background cell populations and limiting the area of microscopic evaluation by using the Centrifuge Chip to create concentrated and low background cell smears. In all samples, malignant and mesothelial cells are found amongst a cellular background of red and white blood cells in standard cytology slides while there are few background cells observed in the Centrifuge Chip-prepared sample slides (**Figure 4A–F**). As expected from our cell measurements above, we concentrated mesothelial and malignant cells in samples diagnosed as positive for malignancy (**Figure 4A–C**). Malignant cells are characterized by large nuclei and high nuclear-cytoplasmic ratio. Malignant cells are often seen as cell aggregates or clumps in effusions [1] and these cell populations were also collected with the Centrifuge Chip. The Centrifuge Chip may aid pathologists in rapid visualization of rarer malignant cells which may improve diagnostic sensitivity especially by enabling processing of larger volumes of fluid into a minimal final concentrated sample volume.

### Effect of Purity on KRAS Gene Mutation Detection

Non-specific amplification from background cells can reduce confidence when measuring the presence of mutations. Pure populations of 10^5^ A549 cells which contain the KRAS* mutation and HeLa cells which have wild type KRAS were measured to have threshold cycles (Ct) of 24.10 and 32.53 respectively (the latter value indicating nonspecific amplification for wild type KRAS from HeLa cells). In mixed samples of A549 and HeLa we found that the presence of the specific KRAS mutation could be distinguished from background at as low as 0.1% purity of A549 cells, with as few as 10 A549 cells present (Figure S3). Note that the same Ct values can be observed from low numbers of A549 cells with specific amplification occurring, or large numbers of HeLa cells with non-specific amplification (see 10,000 HeLa cells vs. 10 A549 cells, **Figure S3A**). Therefore, we normalized the data to account for cell number by subtracting GAPDH Ct from KRAS Ct values, yielding a KRAS* ΔCt. As expected, increased purity samples yielded improved results, characterized by a lower ΔCt (**Figure S3B**).

**Figure 5:**
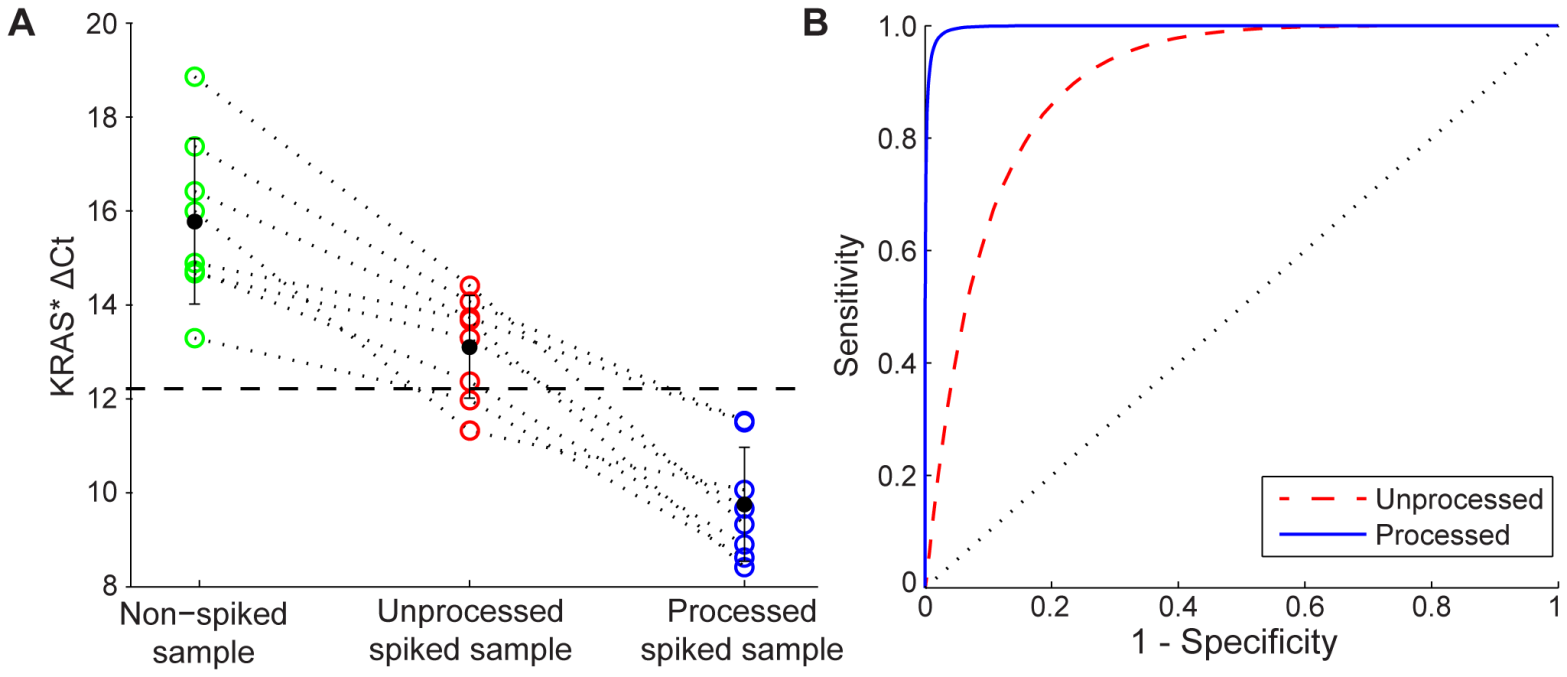
Enhanced RT-PCR Detection Confidence Using the Centrifuge Chip. (A) RT-PCR was performed on non-spiked, unprocessed spiked samples (0.1% purity), and processed spiked samples. KRAS* ΔCt decreased in all cases (n = 7), demonstrating an improved signal for the KRAS mutation. Compared to non-spiked samples, processed spiked samples exhibit a clearer KRAS* ΔCt which is more distinct than for unprocessed spiked samples. The dotted black line shows a 12.2 Ct cut-off threshold for the KRAS mutation. (B) Receiver operating characteristic curves are plotted based on a Gaussian distribution of samples shown in part A. Processed samples show higher area under curve (AUC) values at 0.998 (blue, solid), compared with 0.905 (red, dashed) for unprocessed samples.

The Centrifuge Chip also improved the sensitivity and specificity in detecting A549 cells spiked into negative clinical effusion samples at 0.1% purity (**Figure 5**). Unspiked negative samples (including acute and chronic inflammation samples) averaged a KRAS* ΔCt of 15.7±1.76 (N = 7), and spiked samples at 0.1% purity averaged 12.8±1.39. Once processed with the Centrifuge Chip, the KRAS* ΔCt decreased and became further differentiated from the negative samples in all cases (**Figure 5A**) with an average of 9.6±1.19 ΔCt. Average GAPDH Ct values were 17.63±2.10, 17.80±2.05, and 23.75±2.03 for negative samples, unprocessed spiked samples, and processed spiked samples, respectively. A paired, t-test between non-spiked and spiked samples demonstrated improved statistical significance in the difference in the average KRAS* ΔCt after spiked samples were processed with the Centrifuge Chip (p = 0.0027 before and p = 1.44e−6 after processing). Moreover, using a Gaussian distribution fit to ΔCt values for each group of samples, receiver operating characteristic curves (**Figure 5B**) demonstrated improved area under curve (AUC) values from 0.905 (unprocessed spiked samples) to 0.998 (processed spiked samples). The upper cutoff threshold for a positive KRAS* ΔCt diagnosis was determined by maximizing both sensitivity and specificity, and it was found to be 14.1 for unprocessed and 12.2 for processed samples. By increasing sample purity with the device, we are able to improve KRAS mutation detection and diagnostic confidence.

For a highly specific assay like PCR, our simple concentration approach has the potential to improve diagnostic accuracy for mutational detection when the original mutation is known (e.g., in a sequenced tumor). We expect our technique will be particularly useful in less specific assays, such as gene sequencing, if a particular gene mutation is suspected but the source unknown. As next generation sequencing technologies improve, it may even be possible to do whole transcriptome sequencing, and achieving high purity using an approach such as this would be essential to detect mutations of interest while suppressing non-specific wild-type reads.

## Conclusions

We have developed the Centrifuge Chip, a microfluidic device which can rapidly isolate larger, potentially malignant cells from pleural effusions in a label-free manner with high purity, using size as a biomarker. This chip has several advantages over currently available techniques including speed, robust operation, and ability to process large volumes of sample and concentrate cells into a small end volume. We were able to prepare effusion specimens in ten minutes, an order of magnitude faster than other similar techniques, with increased purity. We also demonstrated that processing by the chip provides improved accuracy detection of mutations with qPCR. This system allows for rapid purification and isolation of cells of interest and has the potential to enable cytopathologists, clinicians, and researchers access to purified cells for preparing cytology slides, detecting specific gene mutations for targeted drug therapies, culturing cells for further analysis, or even isolating of single cells for next generation sequencing analysis at lower cost than currently available techniques. Improved mutational detection at lower cost from readily available body fluids provides a compelling route towards making targeted anti-cancer therapies a broad clinical reality.

## Supporting Information

Figure S1 **Centrifuge Chip System Schematic and Operations.** Sample processing is controlled using an automated pressure system comprised of an air tank, pressure regulators, air and liquid valves, and a computer with a LabVIEW (National Instruments) user interface. A liquid valve upstream from the device switches between the saline wash and pleural sample bottles, and the downstream valve directs fluid between the waste and collection containers. The procedure involves three key steps, including: i.) processing the fluid sample to capture potential cancer cells, ii) washing the device reservoirs to remove smaller leukocytes and RBCs while maintaining the same flow rate and active microvortices to keep larger cells trapped, and iii) lowering the flow rate to release the captured cells from the vortices and into a 96-well plate.
(TIF)

Figure S2 **Sample Processing Flow with the Centrifuge Chip.** 50 mL of pleural effusion sample were processed using traditional cytological methods and the Centrifuge Chip. A portion of cells harvested from the Centrifuge Chip was returned to the cytopathology laboratory to create cell smears; the other portion of processed sample was immunolabeled for purity analysis.
(TIF)

Figure S3 **Effect of cell number and purity on PCR.** (A) Quantitative RT-PCR was performed on cell lines with varying cell number. Ct values for KRAS* (solid line) and GAPDH (dotted line) decreased with increasing cell number. KRAS* Ct for samples with 1,000 HeLa cells or fewer was not detected. (B) KRAS* ΔCt decreases with increasing purity of A549 cells spiked in a larger population of HeLa cells.
(TIF)

Video S1 **Microfluidic processing of a pleural effusion.** A real-time video is shown for Centrifuge Chip processing of a bloody patient pleural effusion specimen. The microfluidic chip is first primed with an isotonic solution, and the automated pressure setup and computerized system controls i) patient sample infusion, ii) solution exchange, and iii) cell release. When the sample is initially infused, the flow rate increases until vortices develop, which is apparent when cells begin to occupy two lateral vortices beside the central flow stream. Next, an upstream valve switches the injection fluid from the specimen bottle to the isotonic wash bottle. During the solution exchange, the remaining small blood cells are observed to wash away, leaving only the stably trapped large cells. Finally, cells are collected off-chip by lowering the wash flow rate to dissipate the vortices and release the cells. The process is repeated as necessary to collect more cells or to process the entire patient sample. Sample infusion time is adjusted for each specimen so that microvortices do not become over-saturated with cells prior to release; infusion times used in this study ranged from 10 seconds to 3 minutes, depending on the sample cellularity.
(WMV)

Table S1 **Complete Summary of 115 Patient Pleural Fluids Used in the Study.** Pos =  positive for malignancy, Sus =  suspicious for malignancy, N =  negative for malignancy, R =  reactive changes, L =  lymphocytosis, CI =  chronic inflammation, and AI =  acute inflammation. Purity is defined as the number of CK+/DAPI+ cells over the total number of cells.
(DOC)
