# Supplementary figures and images for: Microfluidic Purification and Concentration of Malignant Pleural Effusions for Improved Molecular and Cytomorphological Diagnostics
Source: PLoS One. 2013 Oct 28;8(10):e78194. doi: 10.1371/journal.pone.0078194 (PMC3810139; doi:10.1371/journal.pone.0078194)

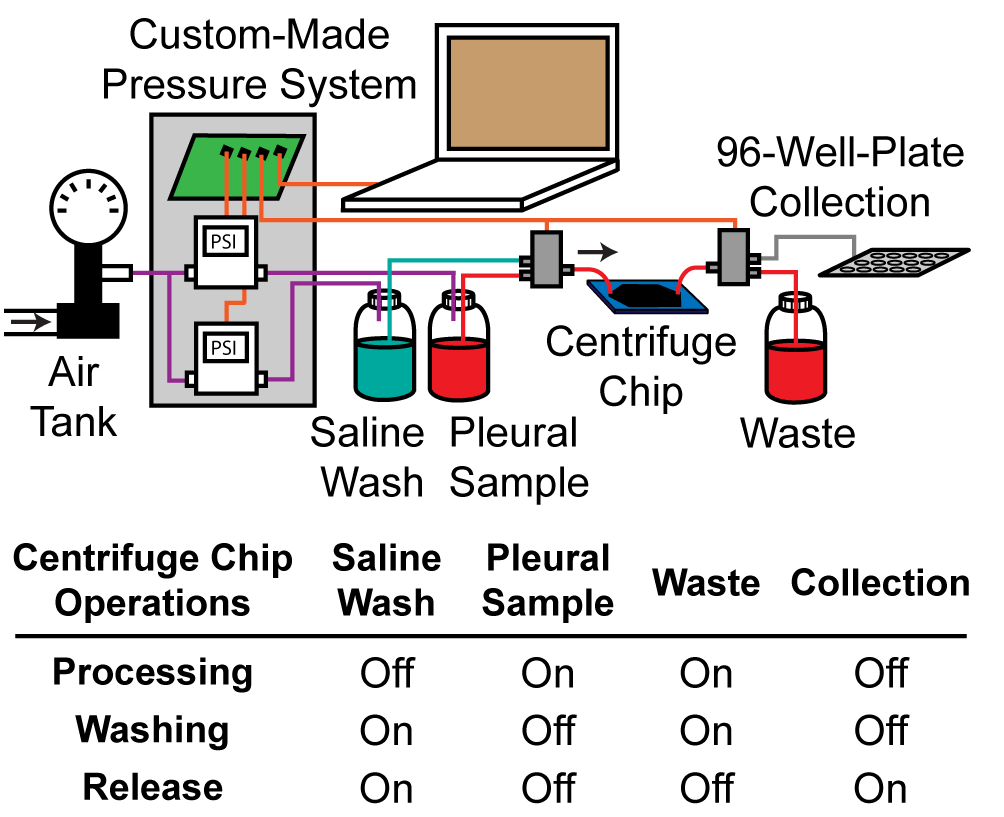

Supplement: Figure S1 — Centrifuge Chip System Schematic and Operations. Sample processing is controlled using an automated pressure system comprised of an air tank, pressure regulators, air and liquid valves, and a computer with a LabVIEW (National Instruments) user interface. A liquid valve upstream from the device switches between the saline wash and pleural sample bottles, and the downstream valve directs fluid between the waste and collection containers. The procedure involves three key steps, including: i.) processing the fluid sample to capture potential cancer cells, ii) washing the device reservoirs to remove smaller leukocytes and RBCs while maintaining the same flow rate and active microvortices to keep larger cells trapped, and iii) lowering the flow rate to release the captured cells from the vortices and into a 96-well plate. (TIF) [file pone.0078194.s001.tif]

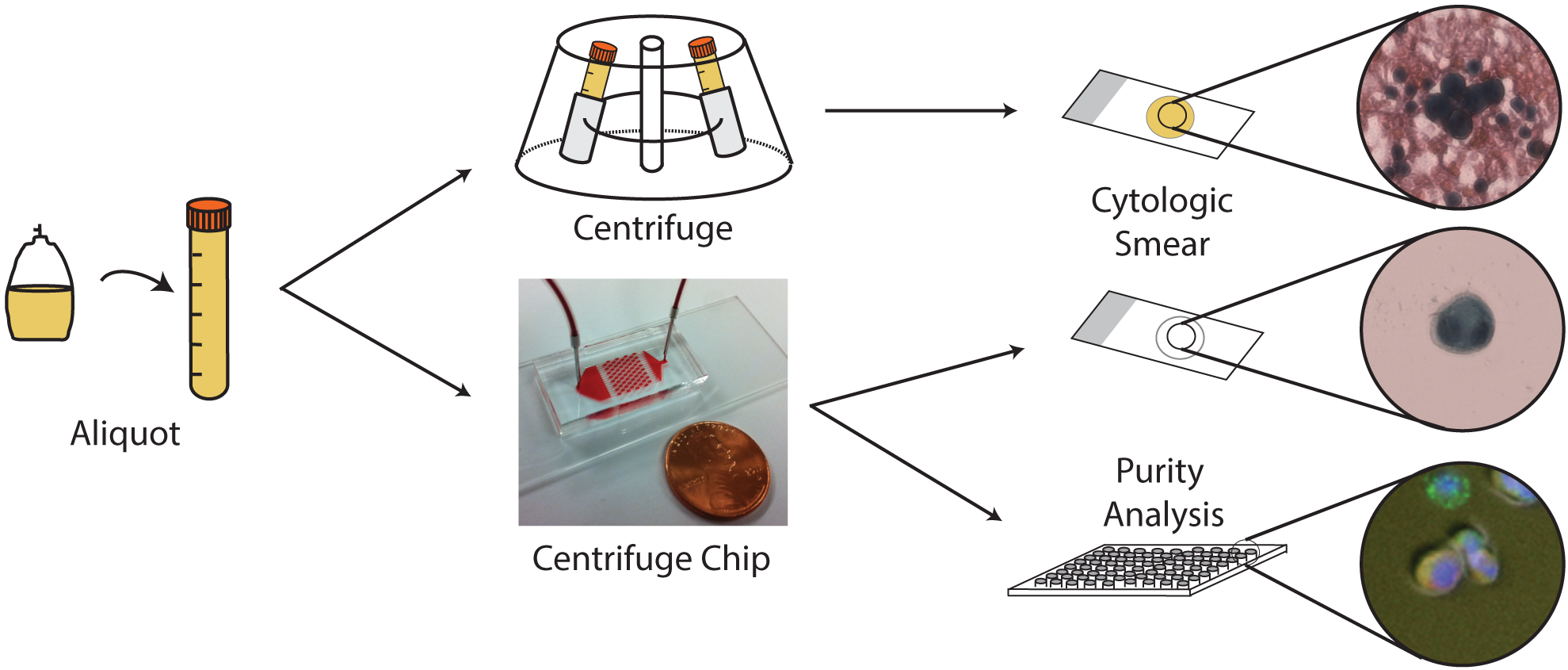

Supplement: Figure S2 — Sample Processing Flow with the Centrifuge Chip. 50 mL of pleural effusion sample were processed using traditional cytological methods and the Centrifuge Chip. A portion of cells harvested from the Centrifuge Chip was returned to the cytopathology laboratory to create cell smears; the other portion of processed sample was immunolabeled for purity analysis. (TIF) [file pone.0078194.s002.tif]

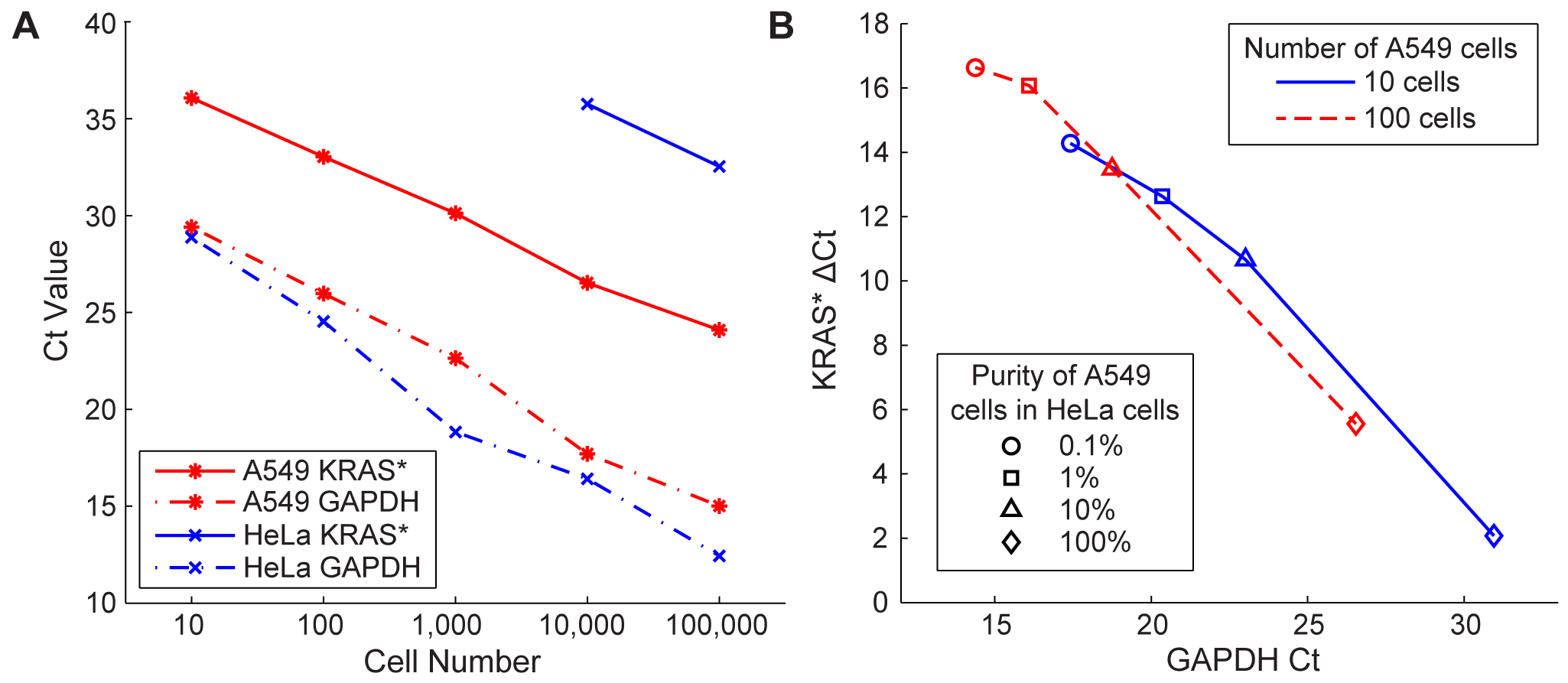

Supplement: Figure S3 — Effect of cell number and purity on PCR. (A) Quantitative RT-PCR was performed on cell lines with varying cell number. Ct values for KRAS* (solid line) and GAPDH (dotted line) decreased with increasing cell number. KRAS* Ct for samples with 1,000 HeLa cells or fewer was not detected. (B) KRAS* ΔCt decreases with increasing purity of A549 cells spiked in a larger population of HeLa cells. (TIF) [file pone.0078194.s003.tif]
