# Supplementary material for: Microfluidic Purification and Concentration of Malignant Pleural Effusions for Improved Molecular and Cytomorphological Diagnostics
Source: PLoS One. 2013 Oct 28;8(10):e78194. doi: 10.1371/journal.pone.0078194 (PMC3810139; doi:10.1371/journal.pone.0078194)
Supplement: Table S1 — Complete Summary of 115 Patient Pleural Fluids Used in the Study. Pos = positive for malignancy, Sus = suspicious for malignancy, N = negative for malignancy, R = reactive changes, L = lymphocytosis, CI = chronic inflammation, and AI = acute inflammation. Purity is defined as the number of CK+/DAPI+ cells over the total number of cells. (DOC) [file pone.0078194.s005.doc]

| **Patient No.** | **Diagnosis** | **Detail** | **No. of CK+ Cells captured** | **Purity (%)** |
| --- | --- | --- | --- | --- |
| 1 | Sus |  | 181 | 58.96 |
| 2 | N | CI | 21 | 95.45 |
| 3 | N | AI |  |  |
| 4 | N | CI |  |  |
| 5 | Pos | Gastric | 1110 | 46.69 |
| 6 | Sus | R | 900 | 28.52 |
| 7 | Pos | Gastric | 23 | 31.94 |
| 8 | N |  |  |  |
| 9 | Pos | Lung | 4800 | 4.05 |
| 10 | Pos | Ovarian |  |  |
| 11 | Sus | R | 31 | 5.86 |
| 12 | Pos | Lung | 6 | 0.16 |
| 13 | N | AI |  |  |
| 14 | N |  | 52 | 21.4 |
| 15 | N |  | 33 | 5.95 |
| 16 | Pos | Lung |  |  |
| 17 | Pos | Breast |  |  |
| 18 | N | CI |  |  |
| 19 | N | CI | 46 | 8.2 |
| 20 | N |  |  |  |
| 21 | N |  |  |  |
| 22 | N | CI | 21 | 33.33 |
| 23 | N |  |  |  |
| 24 | N |  | 75 | 41.9 |
| 25 | N | CI | 114 | 53.27 |
| 26 | Pos | Lung |  |  |
| 27 | Pos | Breast |  |  |
| 28 | N | CI | 39 | 28.06 |
| 29 | N |  |  |  |
| 30 | Pos | Lung |  |  |
| 31 | Sus |  |  |  |
| 32 | N |  |  |  |
| 33 | Pos | Lung | 194 | 49.49 |
| 34 | N |  | 12 | 17.91 |
| 35 | Pos | Breast |  |  |
| 36 | N |  |  |  |
| 37 | N | CI | 12 | 27.91 |
| 38 | N | L | 6 | 6 |
| 39 | N |  | 4 | 20 |
| 40 | N |  | 19 | 36.54 |
| 41 | Pos | Pancreatic |  |  |
| 42 | N | AI | 2080 | 36.24 |
| 43 | N | CI | 64 | 14.61 |
| 44 | N | CI | 131 | 14.46 |
| 45 | N |  | 19 | 7.28 |
| 46 | Pos | Mesothelioma |  |  |
| 47 | Pos | Breast |  |  |
| 48 | N |  |  |  |
| 49 | N | R | 3 | 9.38 |
| 50 | Sus |  | 6 | 6.52 |
| 51 | N | L | 17 | 2.26 |
| 52 | N | CI | 1 | 0.81 |
| 53 | N |  |  |  |
| 54 | N | CI | 32 | 15.09 |
| 55 | N | AI | 12 | 8.89 |
| 56 | Pos | Lung |  |  |
| 57 | N | R |  |  |
| 58 | N |  | 17 | 1.9 |
| 59 | N | CI | 38 | 3.69 |
| 60 | N | CI | 35 | 12.46 |
| 61 | N | R |  |  |
| 62 | Sus |  | 109 | 45.61 |
| 63 | Pos | Esophageal | 7 | 13.46 |
| 64 | N |  |  |  |
| 65 | N |  |  |  |
| 66 | N |  |  |  |
| 67 | N |  | 23 | 8.91 |
| 68 | N |  |  |  |
| 69 | N |  | 15 | 7.35 |
| 70 | N | AI |  |  |
| 71 | N | CI |  |  |
| 72 | N |  | 98 | 4.06 |
| 73 | N | CI | 754 | 69.94 |
| 74 | Pos | Breast | 15 | 83.33 |
| 75 | N |  |  |  |
| 76 | N | C | 129 | 60 |
| 77 | N | AI | 57 | 12.75 |
| 78 | N | C | 253 | 18.32 |
| 79 | N | AI |  |  |
| 80 | N | CI |  |  |
| 81 | N | C | 53 | 22.36 |
| 82 | Sus | CI | 1792 | 33.73 |
| 83 | Sus |  | 107 | 97.49 |
| 84 | N | R |  |  |
| 85 | N | R | 1600 | 87.72 |
| 86 | N | CI | 256 | 20.51 |
| 87 | N |  | 768 | 51.06 |
| 88 | N | R |  |  |
| 89 | N | CI |  |  |
| 90 | N | CI |  |  |
| 91 | Pos | Lung | 25 | 40.98 |
| 92 | N | AI | 576 | 46.15 |
| 93 | Sus | L | 171 | 20.25 |
| 94 | Sus |  | 1024 | 45.71 |
| 95 | Pos | Lung | 53 | 76.81 |
| 96 | Pos | Ovarian |  |  |
| 97 | N | CI |  |  |
| 98 | Pos | Lung |  |  |
| 99 | Pos | Ovarian |  |  |
| 100 | N | L | 145 | 9.15 |
| 101 | Pos | Breast |  |  |
| 102 | N | AI | 101 | 51.79 |
| 103 | Sus | R | 50 | 23.81 |
| 104 | N |  |  |  |
| 105 | N |  |  |  |
| 106 | Sus | R | 44 | 84.62 |
| 107 | N |  |  |  |
| 108 | N |  |  |  |
| 109 | Sus | L | 36 | 28.13 |
| 110 | Pos | Pancreatic |  |  |
| 111 | N | L | 15 | 34.88 |
| 112 | N | CI |  |  |
| 113 | N |  |  |  |
| 114 | N | CI |  |  |
| 115 | N | CI |  |  |
